# Influence of inorganic and organic salts on the hydration mechanism of montmorillonite based on molecular simulation

**DOI:** 10.1038/s41598-023-36137-w

**Published:** 2023-06-05

**Authors:** Lianjun Yang, Bo Xiang, Haisong Zhao, Kai Wu, Enlong Liu, Ge Zhang

**Affiliations:** 1 State Key Laboratory of Hydraulics and Mountain River Engineering, College of Water Resource and Hydropower, Sichuan University, Chengdu, 610065 China; 2 Sichuan Provincial Transport Department Highway Planning, Survey, Design and Research Institute, Chengdu, 610041 Sichuan China; 3 Key Laboratory of Geological Hazards On Three Gorges Reservoir Area, Ministry of Education, China Three Gorges University, Yichang, 443002 Hubei China

**Keywords:** Civil engineering, Structural materials

## Abstract

The molecular dynamics method is used to further reveal, from the molecular point of view, the mechanisms of salt inhibiting the hydration of Na-MMT. The interaction between water molecules, salt molecules, and montmorillonite are calculated by establishing the adsorption models. According to the simulation results, the adsorption conformation, interlayer concentration distribution, self-diffusion coefficient, ion hydration parameters, and other data are compared and analyzed. The simulation results show that the volume and basal spacing increase in a stepwise manner with the increase of water content, and water molecules have different hydration mechanisms. The addition of salt will enhance the hydration properties of compensating cations of montmorillonite and affect the mobility of particles. The addition of inorganic salts mainly reduces the adsorption tightness between water molecules and crystal surfaces, thereby reducing the thickness of water molecules layer, while the organic salts can better inhibit migration by controlling interlayer water molecules. The results of molecular dynamics simulations reveal the microscopic distribution of particles and the influence mechanism when the swelling properties of montmorillonite are modified by chemical reagents.

## Introduction

Montmorillonite has good physical and chemical properties such as adsorption, water absorption, and swelling. Bentonite, with montmorillonite as its main component, can be treated as adsorbent or purifying agent, and is widely used in various industries^1,2^. However, due to its properties of swelling with water absorption and shrinking with water loss, this type of bentonite also poses many engineering problems^3,4^ with water infiltration or evaporation. Even though a great deal of research work has been carried out by many scholars^5–8^, the hydration mechanism of these kinds of materials is still an open issue. Compared with traditional tests, the molecular simulation takes into account theoretical analysis in the microscopic scale, it therefore can not only help to explain the results of conventional experimental studies, but also provide a thermodynamic explanation of the clay swelling mechanism.

Several scholars have now analyzed the water absorption properties of montmorillonite using the molecular dynamics (MD) methods. Wang et al.^9^ simulated the adsorption of water molecules by Li-, Na-, and K-MMT, and showed some differences in the water absorption capacity of montmorillonite with different interlayer exchange cations. Na et al.^10^ used the Cerius to simulate Na-MMT and Na-/Mg-MMT with mixed compensating ions, investigating the effect of different compensating ions on the swelling properties. Huang et al.^11^ investigated the mechanism of inhibiting the hydration of montmorillonite by small organic amine molecules, and found that formamide has a better inhibition effect. Zhao et al.^12^ comprehensively compared the effect of cation species on the diffusion of interlayer particles, showing that certain chemicals containing K^+^ and Ca^2+^ can reduce the swelling properties, which can be used to improve swelling soils. Luo^13^ has studied the inner/outer surface hydration of Na-MMT and showed how some inhibitors work from a microscopic point of view.

However, previous studies on montmorillonite-water-ion system have tended to focus on montmorillonite systems with different compensating cations, whereas natural montmorillonite mineral layers usually contain a variety of cations^14^. The research on the influence of salt solution on the swelling is also focused on the comparative analysis of the type and concentration of cations, while the research on the mechanism and law of the influence of organic salts, especially the functional groups in organic salts, is relatively few. The difference between organic salts and inorganic salts in inhibiting swelling is still unclear. Therefore, there is a great need to further investigate the swelling characteristics of montmorillonite systems with different interlayer salts, to provide a reference for further understanding on the swelling soil improvement using organic matter or chemical solutions to replace exchange cations.

In this paper, a molecular dynamics-based simulation method is used to establish a Na-MMT model to simulate its water absorption and swelling phenomenon. The influence of geometric optimization on the system structure is analyzed, with the aim of studying the swelling behavior of Na-MMT with different types and concentrations of interlayer salts under various water content conditions. Based on the simulation results, the comparisons on the microscopic distribution and diffusion characteristics of interlayer cations and water molecules are also carried out. This research work discusses the mechanism and nature of interlayer particle activities of montmorillonite from a microscopic perspective, which helps to understand the swelling mechanism of swelling clay in more detail, and provides some theoretical references to solve the swelling hazards of clay. At the same time, it compares the differences of mechanism of organic salt and inorganic salt in inhibiting swelling, and better provides the basis for bentonite improvement.

## Model construction and simulation methods

### Na-MMT crystal structure

Montmorillonite is a layered silica-aluminate with a dioctahedral structure, the unit cell of which consists of two silica-oxygen tetrahedral wafers and an aluminium-oxygen octahedral wafer sandwiched between them. The cusps of the silicon-oxygen tetrahedra point towards the aluminium-oxygen octahedra, and the octahedra are connected to the upper and lower tetrahedra by shared oxygen and hydroxide atom clusters to form a tightly packed crystal layer, with the cells connected by an oxygen layer. MMT belongs to the group of 2:1 layered swelling clay minerals.

As the upper and lower outer surfaces of MMT crystals are all layers of oxygen atoms, there is no hydrogen bonding between the MMT crystal layers, and they are combined by intermolecular forces. The attraction between the crystal layers is small, the linkage is weak, and the distance between them is large, so water molecules can easily enter between the two crystal layers to cause swelling. This mineral has a strong lattice substitution phenomenon so that the crystal is negatively charged as well as highly charged, and able to adsorb more cations with a strong ion exchange capacity.

The cell parameters and atomic coordinates of the initial MMT model in this simulation are based on the Wyoming-type cell parameters, which have been widely studied^15,16^. The structure belongs to the monoclinic C2/m space group and symmetrical L^2^PC structure. The lattice constants are α = γ = 90°, β = 99°, a = 0.523 nm, b = 0.906 nm, c = 0.960 nm with no water between crystal layers, and c value varies with the number of water molecules in the interlayer spaces^17^, where a, b, c are the cell axis lengths; the crystal layers extend along the a and b axis directions, stacked along the c axis direction, and (0 0 1) dissociation is complete; and α, β, and γ are the cell axis angles. Considering that the single-cell model does not reflect its periodicity and symmetry, this paper models the system with the periodic boundaries in 8-cells (4a*2b*c), while following Loewenstein's random substitution law^18^, where 1 Al^3+^ is replaced by Mg^2+^ in every 8 aluminium-oxygen octahedra and 1 Si^4+^ in every 32 silicon-oxygen tetrahedra by Al^3+^ substitution. Since the negative charge resulting from the lattice substitution is compensated by Na^+^, the chemical formula can be written as ${\mathrm{Na}}_{6}\left[{\mathrm{Si}}_{62}{\mathrm{Al}}_{2}\right]\left[{\mathrm{Mg}}_{4}{\mathrm{Al}}_{28}\right]{\mathrm{O}}_{160}{\left(\mathrm{OH}\right)}_{32}\cdot {\mathrm{nH}}_{2}\mathrm{O}$, where the SPC/E model^19^ is chosen for the water molecule, that is, the bond distance of an O–H is 1.0 Ǻ and the bond angle of a tetrahedral H–O–H is 109.47°. The water molecules and the clay sheets are considered in a rigid state during the simulation, and the surfactant ions and water molecules are free to move between the two clay layers.

### Simulation methods

Both energy optimization and molecular dynamics simulations of the system are performed through the Forcite module in the Materials Studio software. The UFF force field, which applies to most types of molecular and material research^9,10,12,20^, is employed in the simulation process. In contrast to the Dreiding force field used by Sun et al.^21^, this force field uses the intermolecular static force and van der Waals force to calculate the bonding interactions between atoms on the surface of clay mineral particles, where the non-bonded Coulomb force and van der Waals force are calculated using Ewald and Atom-based summation methods, respectively, with a truncation radius of 9 Å to ensure the long-range electrostatic force accuracy.

The obtained energy minimization model is used as the initial configuration for the molecular dynamics simulation, and all systems are subjected to the NPT ensemble with a time step of 0.5 fs and a system temperature of 298 K. Molecular dynamics simulations are performed for 1000 ps. The first 160 w steps are used to bring the system to equilibrium, and the convergence curves of the system energy changes with computed time are monitored to ensure that the system reaches the equilibrium state, and the statistical average of the last 200 ps is used to calculate the relevant parameters.

## Hydration properties of Na-MMT

### Changes in structure and energy

The structure of Na-MMT adsorbed water molecules is obtained by constructing a Na-MMT supercell in MS and the Sorption module is used to adsorb different amounts of water molecules. However, this structure is not the optimal configuration and requires geometric optimization using the Geometry Optimization function to obtain the optimal configuration by energy minimization.

**Figure 1:**
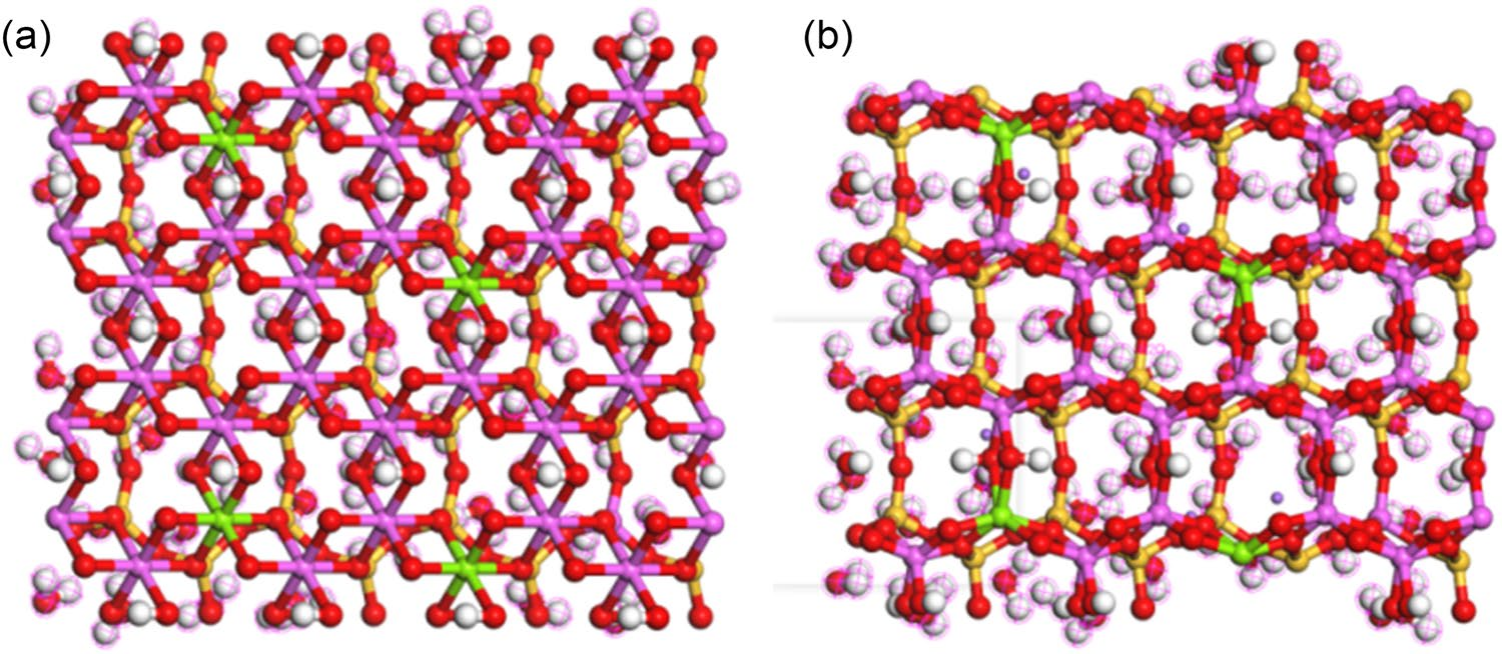
The XOY surface diagrams of Na-MMT adsorption model: (**a**) before optimization; (**b**) after optimization.

**Figure 2:**
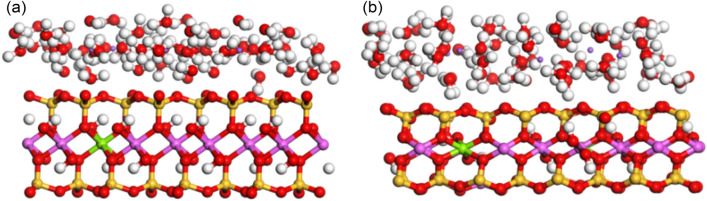
The XOZ surface diagrams of Na-MMT adsorption model: (**a**) before optimization; (**b**) after optimization.

**Figure 3:**
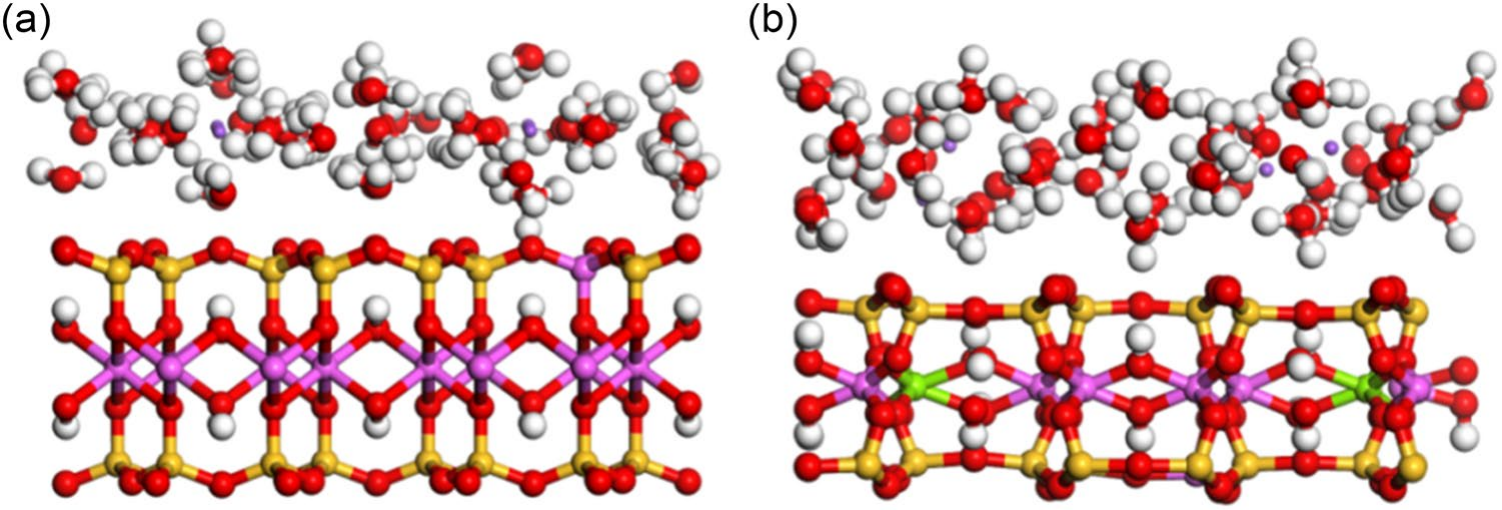
The YOZ surface diagrams of Na-MMT adsorption model: (**a**) before optimization; (**b**) after optimization.

The top view of the XOY surface of the pre- and post- optimization structures of the adsorption 64 water molecules model is shown in Fig. 1, and the side views of the XOZ and YOZ surfaces are shown in Figs. 2 and 3, respectively. By observing and comparing the structures, we can find that: (1) the Na-MMT adsorption model before optimization is in form of regular silicon-oxygen tetrahedra and aluminium-oxygen octahedra, but after optimization the polyhedral structures are distorted; (2) the interlayer cations are regularly arranged in the center of the silicon-oxygen ring before optimization, but after optimization they move closer to the surface of the silicon-oxygen ring and the position of the homogeneous substitution; and (3) the water molecules are more concentrated on the middle surface of the interlayer domain before optimization, after that are distributed in layers. This indicates that the positions of interlayer cations and water molecules in Na-MMT are influenced by the interlayer charge and position.

The energy of the molecular structure system is mainly composed of the bond energy (bond stretching energy, bond angle bending energy, dihedral angle torsion energy) and the non-bond energy (van der Waals interaction energy, electrostatic interaction energy). The changes of energy minimization of the Na-MMT adsorption model are shown in Table 1, and the following conclusions can be drawn: (1) the total energy is significantly reduced after optimization; (2) the bond energy and non-bond energy are reduced to some degree during the optimization process, and the bond stretching energy and the electrostatic energy of the non-bond energy are reduced to a greater extent; and (3) the electrostatic potential energy contributes the most to the total energy before optimization, followed by the bond angle bending energy and the bond stretching energy, after optimization, and the electrostatic potential energy still accounts for the largest proportion, but the proportion of the bond stretching energy becomes smaller.

**Table 1:** Parameters change of energy minimization of Na-MMT.

Number of water molecules	status	Valence (kcal/mol)			Valence (kcal/mol)	Non-bond (kcal/mol)			Non-bond (kcal/mol)	Total (kcal/mol)
		Bonds	Angles	Torsions		vdWs	LR corr	Coulombs		
32	Before	10,360.1	22,746.7	17.6	33,124.3	− 433.7	− 29.0	− 119,055.1	− 119,517.8	− 86,393.5
	After	1063.9	17,991.1	22.9	19,078.0	607.4	− 33.1	− 133,572.1	− 132,997.7	− 113,919.8
	NPT	4478.4	17,502.7	17.4	21,998.5	3303.7		− 145,865.4	− 142,561.7	− 120,563.2
64	Before	10,362.9	22,756.6	17.6	33,137.0	339.9	− 34.0	− 119,379.0	− 119,073.1	− 85,936.0
	After	1068.0	17,995.8	22.9	19,086.8	573.9	− 33.2	− 133,891.5	− 133,350.8	− 114,264.0
	NPT	4248.3	17,286.7	17.9	21,552.9	3171.2		− 145,275.6	− 142,104.4	− 120,551.5
96	Before	10,362.3	22,755.0	17.6	33,134.9	568.0	− 31.8	− 119,594.8	− 119,058.6	− 85,923.7
	After	1074.8	18,031.8	22.8	19,129.4	563.1	− 30.6	− 134,181.7	− 133,649.1	− 114,519.7
	NPT	4248.4	17,320.9	17.0	21,586.2	3082.7		− 145,769.0	− 142,686.3	− 121,100.1

### Expansion curves

**Figure 4:**
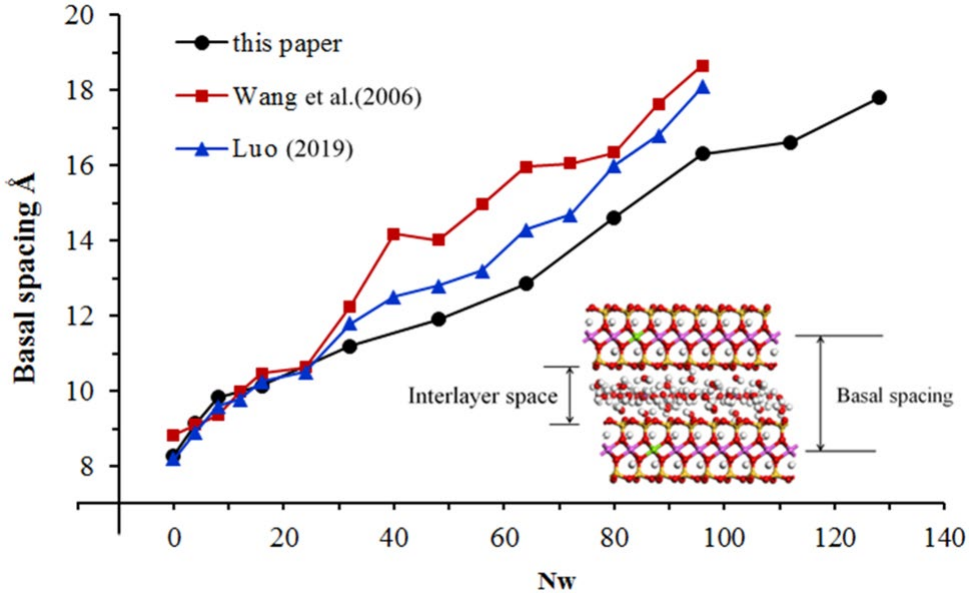
Basal spacing of Na-MMT versus the number of water molecules (Nw).

The layer spacing is an important basis for describing the swelling behavior of clays. From Fig. 4, it can be seen that the Na-MMT layer spacing increases with the number of water molecules in a stepped pattern, which is related to the aggregation of water molecules and the formation of water molecule layers.

The trend of increasing layer spacing is larger for the adsorption of 0–16 and 32–48 water molecules, which corresponds to the beginning of adsorption of the first and second water molecules, respectively. The trend of increasing layer spacing slows down for the adsorption of 16–32 water molecules and 48–80 water molecules, which indicates that the first and second water molecule layers are saturated at this interval. There is no third obvious step in the swelling curve, but to facilitate subsequent analysis, it is considered that Na-MMT forms one, two, and three saturated water molecule layers for adsorbed water molecule numbers of 32, 64, and 96 in combination with the results of X-ray diffraction analysis of Na-MMT^22^.

### Water molecule distribution characteristics

The most important driver of montmorillonite swelling is the interlayer cation hydration, therefore it is crucial to study that. The radial distribution functions and coordination numbers are important methods for analyzing the structure of cation hydration.

#### Radial distribution function

The radial distribution function of a system is the ratio of the density of a spherical region around the given atom i to the average density of the atom j, which is used to describe quantitatively the relative distribution between atoms, and can also be used to describe particle correlations, as defined below.

1 $${\mathrm{g}}_{\mathrm{ij}}\left(\mathrm{r}\right)=\frac{d\mathrm{N}}{4\uppi {\mathrm{r}}^{2}{\overline{\uprho } }_{\mathrm{j}}d\mathrm{r}}$$

where dN is often termed as the coordinate number n(r) for j around i; ${\overline{\uprho } }_{\mathrm{j}}$ is the number density of specie j, and is the average number of the particle of type j lying at the region of r + dr from a particle of type i; and r is the direction from the given atom i.

The radial distribution function g(r) of the components reveals the mode and nature of the interaction between non-bonded atoms. The hydrogen bonding ranges from 0.26 to 0.31 nm, while van der Waals forces range from 0.31 to 0.5 nm, that is, van der Waals forces are weak beyond 0.5 nm, thus the characteristic peaks above 0.5 nm correspond to the long-range intermolecular forces (e.g. electrostatic forces).

**Figure 5:**
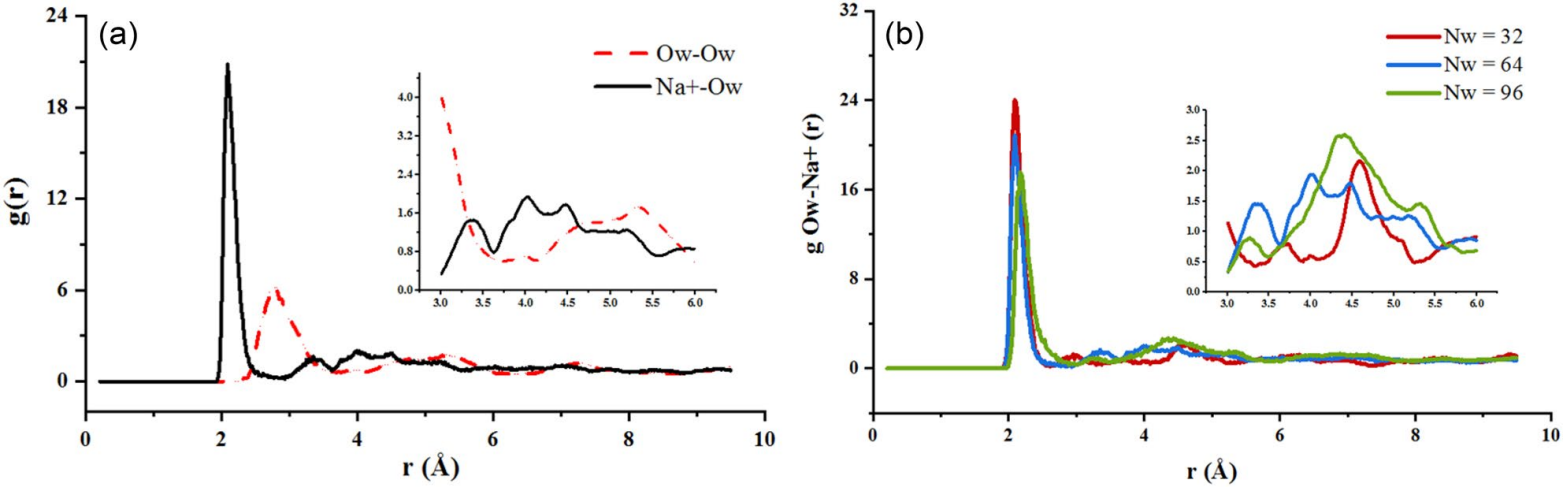
Radial distribution function of Na^+^ in Na-MMT interlayer: (**a**) g_Na+-Ow_(r) and g_Ow-Ow_(r) of 2 w; (**b**) g_Na+-Ow_(r) for different numbers of water molecules.

Some of the radial distribution functions of the simulation are shown in Fig. 5. Analysis shows that: (1) in Fig. 5a, g_Na+-Ow_(r) in all three hydrated states have two peaks with centers near 2.1 Å and 5 Å respectively, and the two peaks of g_Ow- Ow_(r) are significantly lower than those of g_Na+-Ow_(r) for the same number of water molecules; (2) the adsorption distances at the 2 peaks of water molecules and hydrogen atoms are both less than the threshold value of 3.5 Å for the hydrogen bonding association, indicating that the hydrogen bonds are formed between water molecules and clay surface atoms during the adsorption process. The adsorption distance of around 0.99 Å for the main peak is close to the bond length of the hydrogen bonds of water molecules, representing the distribution odds of H bound to O in the same water molecule, and around 2.3 Å for the secondary peak, representing that water molecules form the hydrogen bonds with more water molecules around them through their own O_w_ and H_w_. This effect gradually decreases with increasing distance from the (0 0 1) surface until it reaches the state of free water; and (3) in Fig. 5b, the peak value of the radial distribution function between the same particles differs when the number of water molecules adsorbed varies, indicating that the hydration state of the Na-MMT can affect the structure and particle distribution of water molecules.

#### Ion coordination numbers

The ion coordination number is the number of particles around a particle that is coordinated with it and can be obtained by integration of the radial distribution function, it is defined as:

2 $${\mathrm{n}}_{\mathrm{ij}}\left(\mathrm{r}\right)=4\uppi {\uprho }_{0}{\int }_{0}^{\mathrm{r}}{\mathrm{r}}^{2}{\mathrm{g}}_{\mathrm{ij}}\left(\mathrm{r}\right)\mathrm{dr}$$

where ${\uprho }_{0}$ is the number density and ${\mathrm{g}}_{\mathrm{ij}}\left(\mathrm{r}\right)$ is the radial distribution function of atoms *i* and *j.*

#### The ion hydration number

Particles in solution, whose electrostatic force can affect the structure of nearby water molecules so that the immediately surrounding particles are arranged around the central atom, can move along with the central ion. The calculation of the ion hydration number is related to the calculation of the ion coordination number and it decreases with increasing hydration. The decrease in the hydration number of sodium ions is related to the decrease in the coordination number. It is defined as:

3 $${\mathrm{n}}_{\mathrm{hyd}}={\mathrm{n}}_{\mathrm{ij}}\left(\mathrm{r}\right)\mathrm{h}$$

where ${\mathrm{n}}_{\mathrm{hyd}}$ is the ion hydration number; ${\mathrm{n}}_{\mathrm{ij}}\left(\mathrm{r}\right)$ is the ion coordination number; $\mathrm{h}$ is the ion hydration factor; and the Na cation hydration factor is 0.69.

#### Ion hydration radius

The water molecules surround the particles closely, increasing their volume. We have the following expression:

4 $$\uppi {{\mathrm{r}}_{\mathrm{hyd}}}^{3}=\mathrm{\vartheta }{\mathrm{n}}_{\mathrm{hyd}}+\uppi {{\mathrm{r}}_{\mathrm{eff}}}^{3}$$

where ${\mathrm{r}}_{\mathrm{hyd}}$ is the ion hydration radius, nm; $\mathrm{\vartheta }$ is 2.99 × 10^–29^ m^3^, the volume of water molecules; ${\mathrm{r}}_{\mathrm{eff}}$ is the ion effective radius, defined as the difference between the effective radius of the first peak of the radial distribution function of water, where the effective radius of the water molecule is 0.138 nm^23^.

The ion coordination numbers, hydration numbers, and hydration radius of the Na cations in one, two, and three hydration layers obtained are shown in Table 2. As can be seen from Table 2, the ion coordination number, hydration numbers, and hydration radius of Na cations all decrease with the increase in the number of water molecules in the interlayers, which shows the reduction of the attractive force exerted by Na cations on water molecules. This phenomenon is due to an increase in the number of water molecules, leading to an increase in the spacing of the Na-MMT layers, with Na^+^ gradually being influenced by the electrostatic gravitational force on one side only, as well as a weakening of the Na–O bond formed with the oxygen in the water molecules, and thus resulting a decrease in its ability to aggregate water molecules. Na^+^ loses coordination with water molecules during the hydration process of Na-MMT, that is, the ability to aggregate water molecules gradually decreases.

**Table 2:** Ion hydration number, hydration numbers, and hydration radius of Na^+^ in Na-MMT.

The hydration degree	Ion coordination number	Ion hydration number	Ion hydration radius
1 layer	4.48	3.09	3.10
2 layers	3.28	2.26	2.80
3 layers	3.15	2.17	2.77

#### The mean square displacement curves

The mean square displacement (MSD) is a measure of the deviation of the position of the particle after it has moved over time from the reference position. The self-diffusion coefficients D of the cations and water molecules in the interlayer space can be calculated by the use of the two-dimensional Einstein relation as follows:

5 $$\mathrm{MSD}=\langle {\left|\mathrm{r}\left(\mathrm{t}\right)-\mathrm{r}\right|}^{2}\rangle =\frac{1}{\mathrm{n}}{\sum }_{\mathrm{i}=1}^{\mathrm{n}}{\left|\mathrm{r}\left(\mathrm{m}+\mathrm{i}\right)-\mathrm{r}\left(\mathrm{i}\right)\right|}^{2}$$

6 $$\mathrm{D}=\frac{1}{6\mathrm{t}}\langle {\left|\mathrm{r}\left(\mathrm{t}\right)-\mathrm{r}\right|}^{2}\rangle$$

where $0<\mathrm{m}+\mathrm{n}=\mathrm{k}$, and m is the maximum value allowed for the MSD calculation, n is the number used for averaging, and k is the total number counted. The self-diffusion coefficient is in Å^2^/ps.

**Figure 6:**
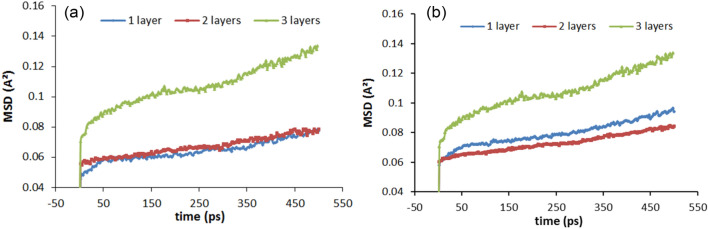
MSD curves of Na cations and water molecules into one, two, and three hydration layers: (**a**) Na cations; (**b**) O atoms.

**Figure 7:**
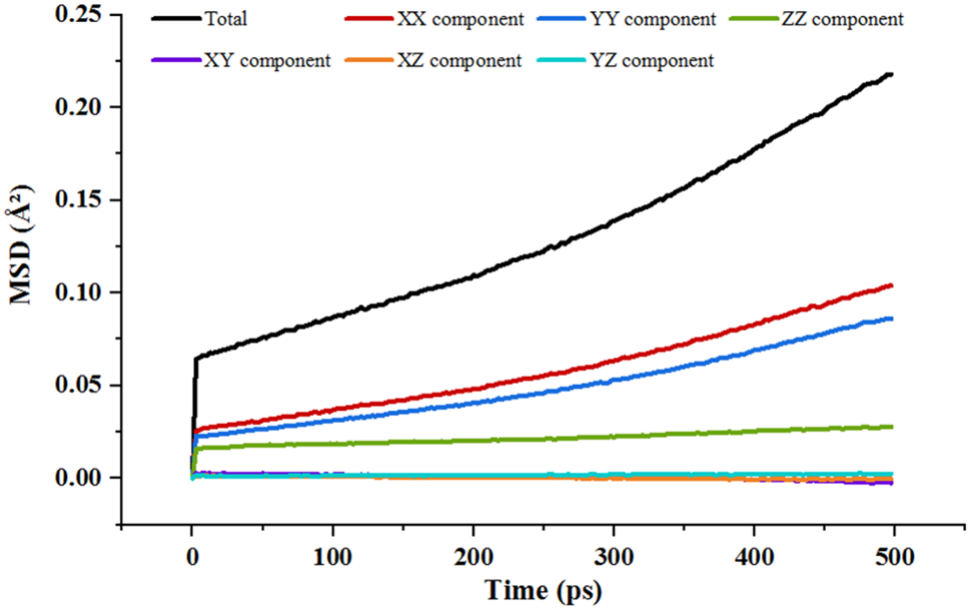
MSD curves of water molecules for 2w Na-MMT with 16.76% ${\mathrm{CH}}_{5}{\mathrm{SiO}}_{3}\mathrm{Na}$.

The mean square displacement curves of Na cations and O_w_ atoms (water molecules) are given in Fig. 6. From Fig. 6, it is observed that the diffusion of Na cations and water molecules into three hydration layers is higher than that of two and one hydration layers, but the MSD curves do not change much with increasing time. The total MSD can decompose into six contributions, namely XX, YY, ZZ, XY, XZ, and YZ component. The mean square displacement curve of water molecules for 2w Na-MMT with 16.76% ${\mathrm{CH}}_{5}{\mathrm{SiO}}_{3}\mathrm{Na}$ is given in Fig. 7, and it shows that the diffusion degree of water molecules in z-direction is very low, and its contribution to the total diffusion is very small. This is due to that the diffusion of interlayer particles in the z-direction is limited by the hydrogen bonding of the clay skeleton as well as the surface-bridging oxygen atoms, which contribute in the z-direction small.

From the table of the self-diffusion coefficients for ions and water molecules in Table 3, it can be seen that the self-diffusion coefficient as a whole shows an increase with increasing water content, with the smallest value at two layers of water molecules. Under the same water content conditions, the mobility of Na cations is lower compared to water molecules, and the Na cations vary at a slower rate than water molecules in different hydration states. The analysis leads to the conclusion that the strong electrostatic interaction between such ions and the charged clay surface significantly slows down the diffusion of metal ions.

**Table 3:** Simulated self-diffusion coefficient of interlayer species of Na-MMT.

The hydration degree	${D}_{{Na}^{+}}$ (10^–13^ m^2^/s)	${D}_{{H}_{2}O}$ (10^–13^ m^2^/s)
1 layer	0.81	0.99
2 layers	0.77	0.78
3 layers	1.66	1.66

## Simulation of cation-inhibited hydration mechanisms

The simulations are carried out with four salt solutions of NaCl, KCl, CH_5_SiO_3_Na, and CH_5_SiO_3_K at different mass fractions, and the Na-MMT in the two-layer hydrated state is used as the base system. The simulations of this part are still conducted in the NPT ensemble with a time step of 0.5 fs and a system temperature of 298 K, which are performed for 1000 ps. The statistical average of the last 200 ps is used to calculate the hydration parameters, self-diffusion coefficients, and other relevant parameters.

**Figure 8:**
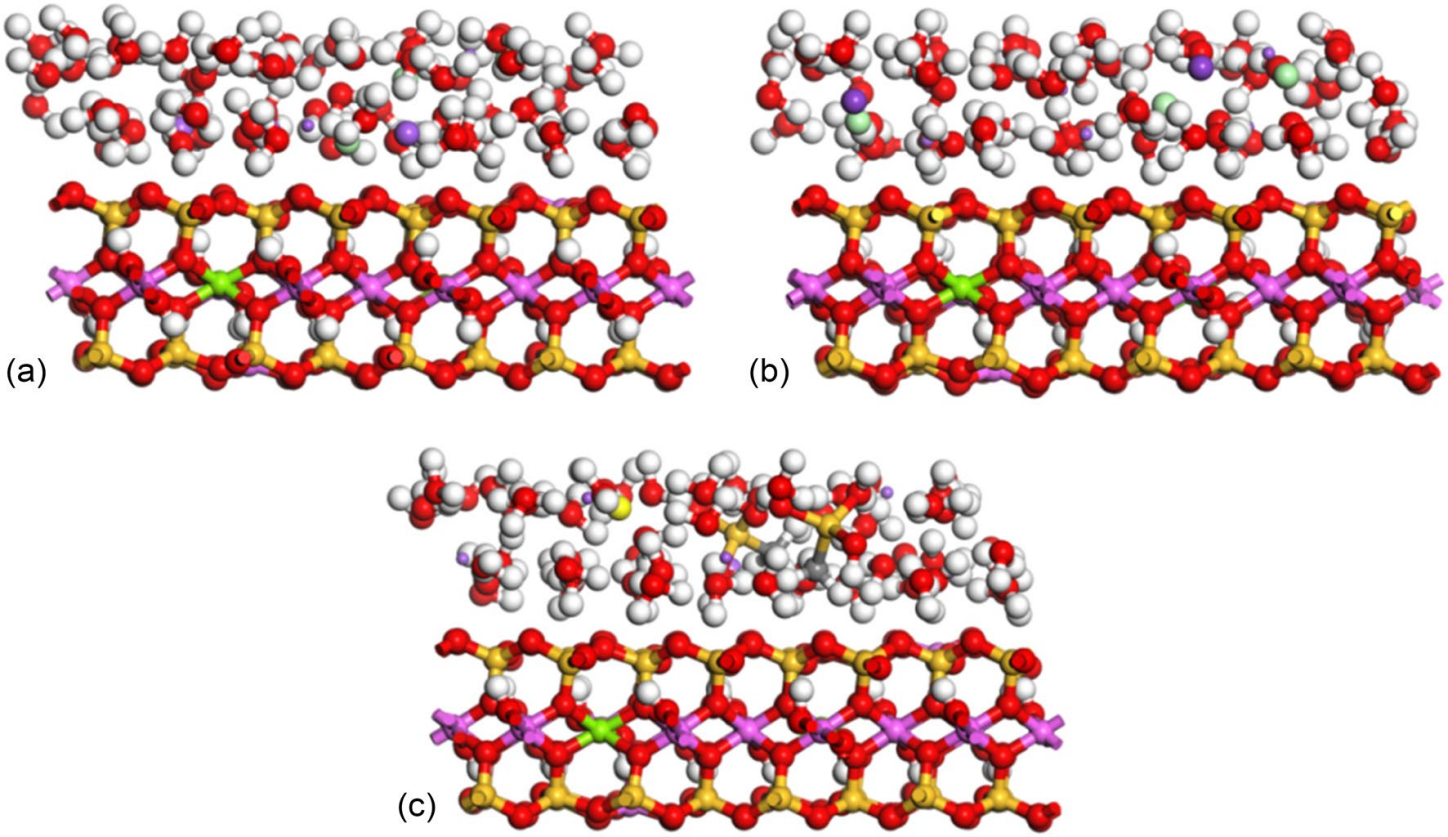
The XOZ surface structure diagrams of 2 layers model after NPT: (**a**) 16.88% NaCl; (**b**) 16.25% KCl; (**c**) 16.76% CH_5_SiO_3_Na.

The side views of the XOZ surface of the structures simulated after NPT ensemble of the 2 layers models with different salt solutions are shown in Fig. 8.

### Effect of inorganic salts addition on the ion parameters

As can be seen from Table 4, we can have the following conclusions: (1) the hydration parameters of Na^+^ all show an increase, then a decrease, and then an increase with the increasing mass fraction after the addition of different types of salts, which is due to the presence of a large number of unabsorbed free water molecules between the layers. In the beginning, as the mass fraction of salts increase, the interlayer ions adsorb free water molecules and the hydration parameters increase. As the mass fraction of salts continue to increase, the amount of free water decreases and the competition between the ions for the adsorption of water molecules occurs. When the mass fraction of salts increases further, the probability of collision between the anions and cations in the interlayer of montmorillonite increases and releases some of the free water for further ion adsorption. Xu et al.^24^ also came to a similar conclusion; (2) Compared to different cationic salts, the parameters are greater with the addition of the same concentration of K-salt than the one of Na-salt. The reason is that K^+^ has less hydration number and a smaller hydration radius, which makes some of those can be embedded in the hexahedral ring enclosed by silica-oxygen tetrahedra and stick to the inner surface of montmorillonite, so that the interlayer force converts from mainly inter-molecular force to mainly electrostatic force. It inhibits the hydration of Na-MMT and reduces the tendency of interlayer expansion. Conversely, Na^+^ can only hang above the tetrahedra due to its higher hydration number and radius, which only serves to weaken the negative charge repulsion between the crystal layers; and (3) the greater hydration parameters of Na^+^ in organic salt simulations compared to inorganic salts may be due to their larger size, which allows them to occupy more space and makes water molecules get closer to the compensating cation.

**Table 4:** Ion hydration number, hydration numbers and hydration radius of Na cations after the addition of salt solutions.

States	Mass fractions (%)	Ion coordination number	Ion hydration number	Ion hydration radius
2 layers	–	3.28	2.26	2.80
2 layers + NaCl	4.83	3.57	2.46	2.88
	9.22	3.27	2.26	2.80
	13.22	3.22	2.22	2.78
	16.88	3.85	2.66	2.95
	20.25	2.95	2.04	2.70
2 layers + KCl	6.07	3.42	2.36	2.84
	11.45	3.50	2.42	2.86
	16.25	3.45	2.38	2.85
	20.55	3.48	2.40	2.85
	24.43	3.38	2.33	2.83
2 layers + CH_5_SiO_3_Na	9.15	3.86	2.66	2.96
	16.76	3.70	2.55	2.91
	23.2	4.04	2.79	3.00
	28.71	3.75	2.59	2.93
	33.49	3.67	2.53	2.91
2 layers + CH_5_SiO_3_K	10.28	3.71	2.56	2.92
	18.64	3.37	2.32	2.82
	25.58	3.48	2.40	2.85
	31.43	4.55	3.14	3.12
	36.42	3.97	2.74	2.98

### Effect of inorganic salts addition on the diffusion

The self-diffusion coefficients of the interlayer particles after the addition of salt are shown in Table 5, from which we can have that: (1) comparing the addition of salt inhibitors, the self-diffusion coefficients are all increased. The cations in the salt interact strongly with the negative charge of the clay sheet rather than with the interlayer water molecules, which leads to a reduction in the attraction exerted by the clay sheet on the interlayer particles and an increase in the mobility of the Na^+^ and water molecules; (2) the migration of Na^+^ and water molecules is higher in the presence of added K-salts, since K cations are less attractive to water molecules than Na^+^, as well as K cations do not interact strongly with interlayer particles; and (3) the overall pattern shows that the self-diffusion coefficient of the interlayer particles increases first and then decreases with the increasing concentration of the added salt inhibitor. When small amounts are added, the cations in the salt attract a small number of interlayer particles, reducing the attraction of the clay sheets to the interlayer particles, and their mobility increases substantially. As more salt cations are added, more interlayer particles are adsorbed and fewer interlayer particles are free to move, that is, the self-diffusion coefficient decreases.

**Table 5:** Self-diffusion coefficient of interlayer species after the addition of salt solutions.

States	Mass fractions (%)	${D}_{{Na}^{+}}$ (10^–13^ m^2^/s_)	${D}_{{H}_{2}O}$ (10^–13^ m^2^/s)
2 layers	–	0.77	0.78
2 layers + NaCl	4.83	0.82	0.8
	9.22	1.13	1.35
	13.22	10.20	2.13
	16.88	0.87	0.68
	20.25	2.72	5.7
2 layers + KCl	6.07	1.61	2.3
	11.45	0.72	1.01
	16.25	1.95	1.08
	20.55	0.58	0.95
	24.43	1.37	1.07
2 layers + CH_5_SiO_3_Na	9.15	16.82	5.43
	16.76	9.96	5.25
	23.2	2.40	2.48
	28.71	2.49	1.47
	33.49	0.65	1.26
2 layers + CH_5_SiO_3_K	10.28	5.43	0.76
	18.64	5.52	1.24
	25.58	2.48	4.98
	31.43	1.47	2.85
	36.42	1.26	1.93

### The relative concentration distribution curve

**Figure 9:**
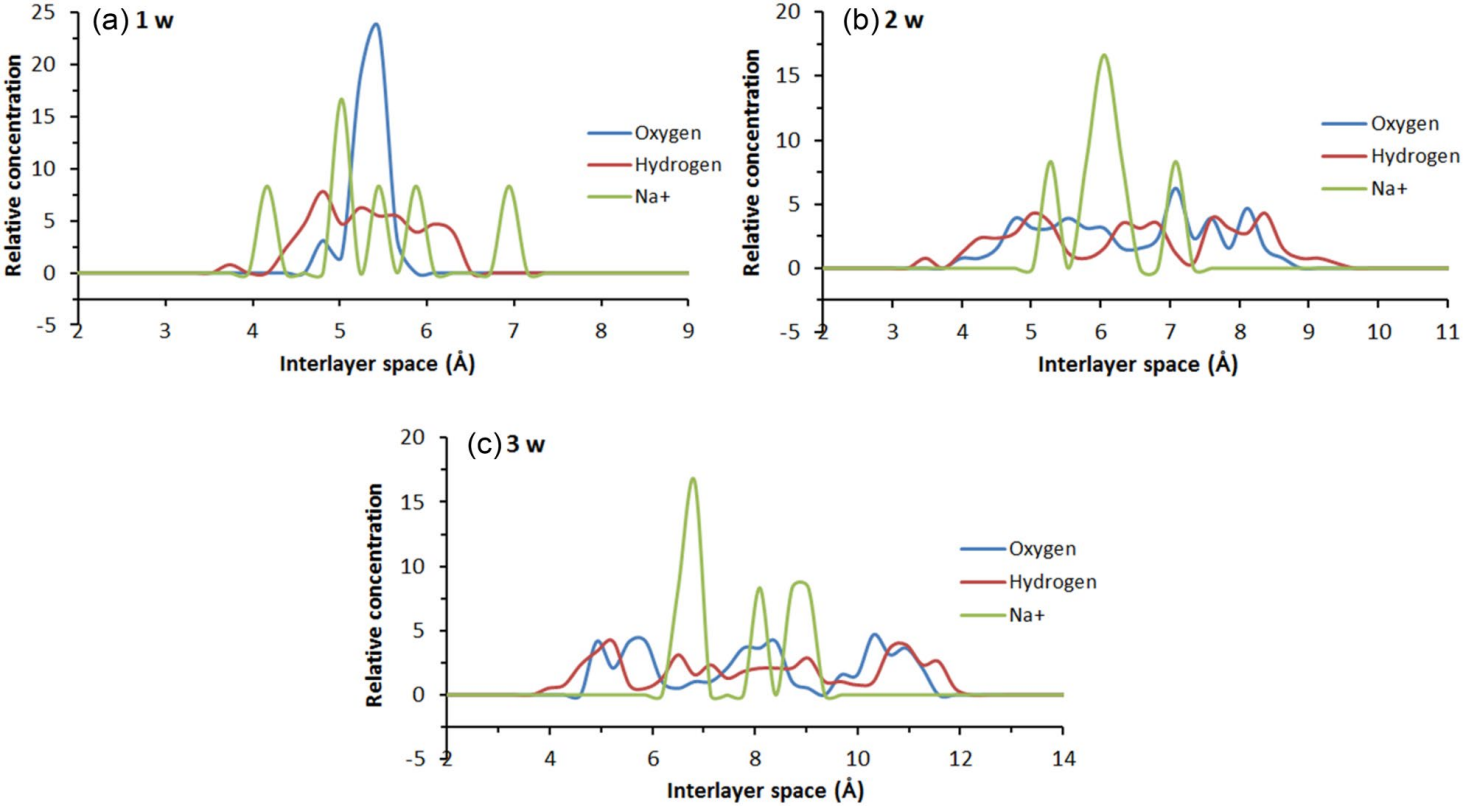
Variation of water and cation relative concentration profile with the interlayer space: (**a**) 1 w; (**b**) 2 w; (**c**) 3 w.

**Figure 10:**
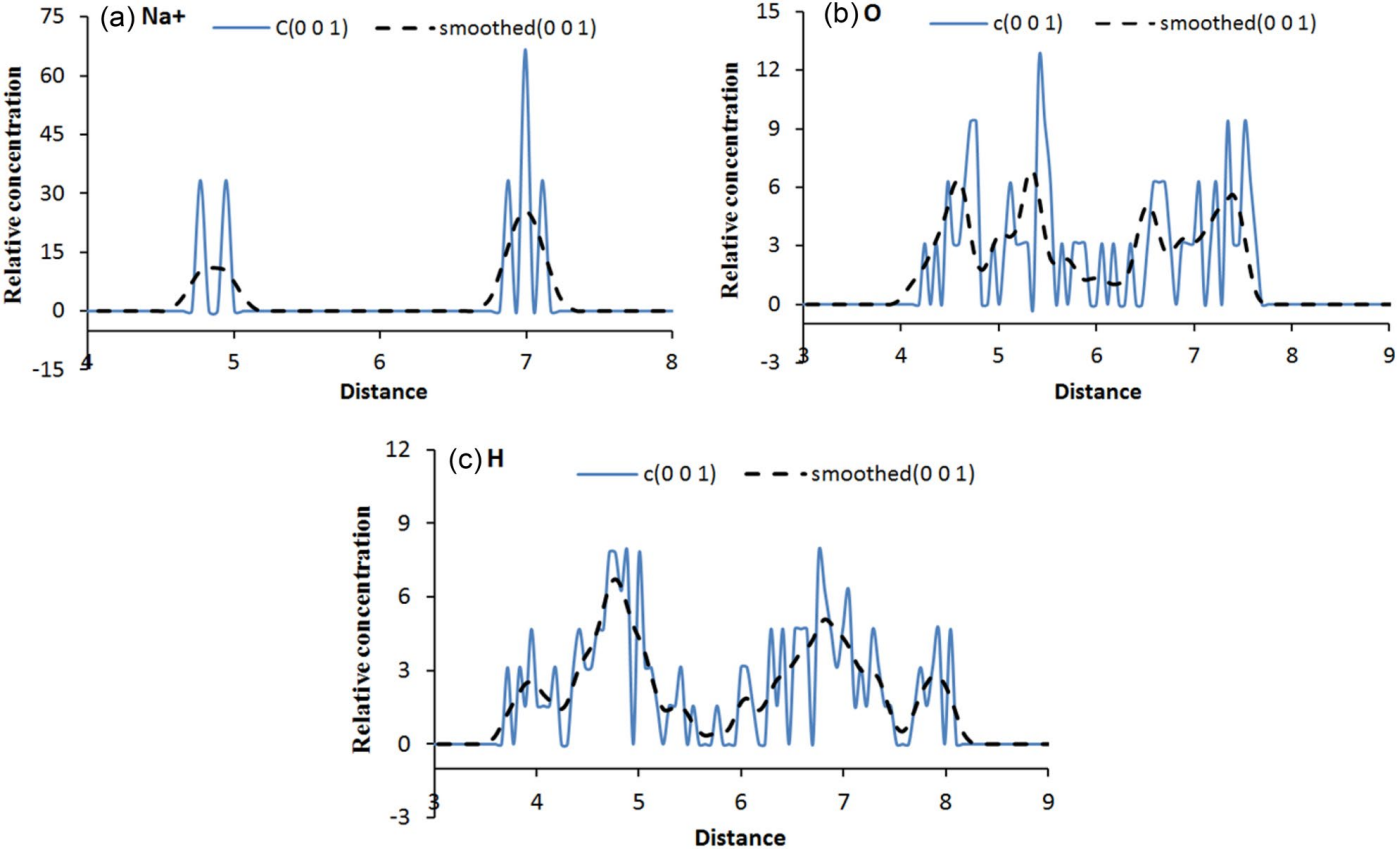
Variation of water and cation relative concentration profile with an interlayer space of 2 w for 9.15% CH_5_SiO_3_Na: (**a**) Na^+^; (**b**) O; (**c**) H.

**Figure 11:**
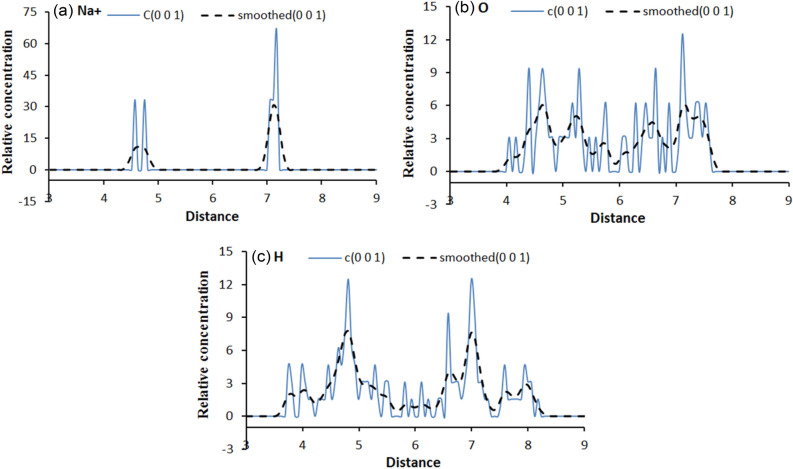
Variation of water and cation relative concentration profile with an interlayer space of 2 w for 10.28% CH_5_SiO_3_K: (**a**) Na^+^; (**b**) O; (**c**) H.

Part of the relative concentration distribution curves along the (0 0 1) direction for hydrogen, and oxygen atoms in the interlayer water molecules as well as the compensating cations are respectively shown in Figs. 9, 10 and 11, and. It can be seen that: (1) in Fig. 9a, the curve of hydrogen atoms in water does not show an obvious unimodal distribution in the formation of the first hydration layer, but the oxygen atoms show a concentrated distribution. Similarly, Na^+^ shows a three-peak distribution and a highest-peak in the middle, which indicates that Na-MMT is still in a one-layer hydration state at the number of interlayer water molecules of 32; (2) with an increase in the number of interlayer water molecules, as shown in Fig. 9b,c, the relative concentration distribution of water molecules and ions changes, showing a multi-peak distribution, with water molecules being more dispersed and symmetrically distributed between the upper and lower layers. The more layers hydrate, the closer to the mineral surface the location of the peak the water molecule concentration distribution corresponds to; (3) in Figs. 10 and 11, taking the 2 w hydrated state as an example, the pattern of the curves after adding different mass fractions of salt is almost the same, all of which show a multi-peak distribution, indicating that the addition of salt solution has little effect on the particle concentration distribution; and (4) under different simulation conditions, the majority of water molecules are distributed around the cations in the interlayer region of the montmorillonite, while Na^+^ is either concentrated in the interlayer region or closer to the clay surface, indicating a wide range of movement.

## Conclusions

In this work, we have simulated the microstructure of the montmorillonite-water-ion system at ambient temperature and pressure to obtain its micromechanical response properties at the molecular level, based on molecular dynamics simulations. The main conclusions are summarized as follows:

1. Montmorillonite hydration is divided into two stages: crystalline swelling and osmotic swelling, where the simulated part is under the crystalline swelling stage. The basal spacing increases in steps with an increasing number of adsorbed water molecules, which are equal to 32, 64, and 96, respectively, and there is a formation of one, two, and three hydration layers.
2. The statistical results of the radial distribution function and coordination number quantitatively characterize the micro-structural differences in Na-MMT at different water contents. Both the increase in water content and the addition of salts enhance the hydration properties of the ions in the system, which explains the mechanism of chemical reagents used to reinforce swelling soils.
3. The self-diffusion of interlayer particles is limited by the surface of the montmorillonite mineral. The mobility of interlayer particles increases with the rise in the hydration degree from one to three hydration layers. And when inorganic or organic salts are added, an increase in the mobility of interlayer particles with NaCl, KCl, CH_5_SiO_3_Na, and CH_5_SiO_3_K addition is observed. The addition of inorganic salts mainly reduces the adsorption tightness between water molecules and crystal surfaces, thereby reducing the thickness of water molecules layer, while the organic salts control the interlayer water molecules. Organic salts have a good inhibition effect on the mobility of Na cations and water molecules at this hydration state.

## Data Availability

Data will be made available from the corresponding author on request.
